# Supplementary material for: Determinants of SARS-CoV-2 infection in Italian healthcare workers: a multicenter study
Source: Sci Rep. 2021 Mar 11;11:5788. doi: 10.1038/s41598-021-85215-4 (PMC7970984; doi:10.1038/s41598-021-85215-4)
Supplement: Supplementary file 1 — Supplementary Information 1. [file 41598_2021_85215_MOESM1_ESM.pdf]

Supplementary Table 1. Availability of information by center

Variable	*Bari*	*Bologna*	*Brescia*	*Genoa*	*Pisa*	*Turin*
<u>Demographics</u>						
Sex	Y	Y	Y	Y	Y	Y
Age	Y	Y	Y	Y	Y	Y
<u>Occupation</u>						
Job title	Y	Y	Y		Y	Y
Resident status				Y		
COVID-19 department	Y	Y	Y			Y
<u>Source of contact</u>						
Contact outside wp		Y	Y		Y	
Contact at wp		Y	Y			Y
Contact with colleague			Y		Y	Y
Contact with patient			Y		Y	Y
<u>Use of PPE</u>						
Surgical mask	Y		Y			Y
FFP2/FFP3 mask	Y		Y			Y
Surgical or FFP mask	Y		Y	Y		Y
Face shield	Y		Y	Y		Y
Gloves	Y		Y	Y		Y
Gown	Y		Y	Y		Y
Any PPE	Y		Y	Y	Y	Y
Mask (contact)						Y
Mask (both HCW and contact)						Y
<u>Symptoms</u>						
Fever			Y	Y	Y	Y
Cough			Y	Y	Y	Y
Dyspnea			Y	Y	Y	Y
Sore throat			Y	Y	Y	Y
Rhinitis			Y	Y		Y
Ageusia or anosmia			Y	Y	Y	Y
Diarrhea			Y	Y		Y
Malaise			Y		Y	
Fever, cough or dyspnea		Y	Y	Y	Y	Y

wp, workplace; PPE, personal protection equipmen; FFP, filtering facepiece
